# Rethinking the Discharge Policy for Ebola Convalescents in an Accelerating Epidemic

**DOI:** 10.4269/ajtmh.14-0719

**Published:** 2015-02-04

**Authors:** Tim O'Dempsey, S. Humarr Khan, Daniel G. Bausch

**Affiliations:** Liverpool School of Tropical Medicine, Liverpool, United Kingdom; Kenema Government Hospital, Ministry of Health and Sanitation, Sierra Leone; Tulane University Health Sciences Center, New Orleans, Louisiana; U.S. Naval Medical Research Unit No. 6, Lima, Peru

## Abstract

The outbreak of Ebola virus disease (EVD) in West Africa has outstripped available resources. Novel strategies are desperately needed to streamline operations. The present norm of requiring negative results on polymerase chain reaction for EVD convalescent patients to be discharged is not evidence-based and often results in asymptomatic patients competing for beds in dangerously crowded Ebola Treatment Units, posing risks to ward staff and patients and the community if infected persons are turned away. We summarize the relevant data and call for a change in discharge criteria for convalescent patients that can safely help reduce the strain on resources and direct energies where they are most needed. In the longer term, research is needed to assess the true infectivity of EVD convalescent patients to establish evidence-based criteria and guidelines for discharge.

The outbreak of Ebola virus disease (EVD) in West Africa has been declared a public health emergency of international concern by the World Health Organization (WHO),^1^ in large part because the scale of the outbreak has outstripped available resources.^2^,^3^ We believe that a change in the discharge policy for convalescent patients can help reduce the strain on resources and direct energies where they are most needed.

In the last 15 years mobile laboratories have been routinely installed in epidemic areas to perform diagnostic testing for EVD by testing of blood by real-time polymerase chain reaction (PCR). With the assay at hand, patient discharge criteria of being asymptomatic for 3 days *and* testing PCR negative has become the norm.^4^ In our experience providing patient care at Ebola Treatment Units (ETUs) in Sierra Leone and Guinea during this outbreak, the interval between a patient becoming asymptomatic and testing PCR negative varies widely. In many cases completely asymptomatic convalescent patients wait days or even weeks to be released, either because their PCR result remains positive, often at a very low level, or simply because the laboratory is too over-run with samples from suspected new cases to perform the assay on the convalescents. Meanwhile, in many ETUs suspected cases are being turned away because of a critical shortage of beds; we faced this situation in the ETU at Kenema Government Hospital, Sierra Leone, in July 2014, when ∼25% of the official bed capacity was occupied by asymptomatic but PCR-positive patients, usually very impatient to go home. Wards became dangerously overcrowded and increasingly hazardous for staff and patients, deterring EVD cases from being admitted to the Kenema ETU, creating a backlog of EVD-confirmed cases in peripheral holding units, which necessitated diversion of cases to the already over-crowded ETU run by Medicine Sans Frontieres in neighboring Kailahun District.

In addition to the operational challenges caused, there is little justification of the PCR-negative requirement on scientific grounds. The viral load in EVD closely parallels the clinical status^5^ and is thus logically very low in asymptomatic persons. In contrast, symptomatic patients, particularly when severely ill, have high viral loads and are very infectious, posing a far greater community transmission threat.^5^,^6^ No secondary transmission was noted in a study of 152 household contacts of 29 convalescent EVD patients followed for up to 21 months after illness.^7^ All 481 specimens (85 tears, 84 sweat, 79 feces, 95 urine, 86 saliva, 8 semen, 44 vaginal secretions) taken from the convalescent patients between Days 12 and 157 after the onset of illness tested negative by both PCR and cell culture, with the exception of a few PCR-positive results from semen, consistent with the gonads being an immunologically protected site where persistence for months after infection has been recognized.^5^,^8^ Furthermore, PCR positivity does not necessarily indicate the presence of infectious virus, but may simply indicate residual nucleic acids being cleared. Cell culture results are very often negative on PCR-positive specimens.^8^,^9^

The epidemic has continued to accelerate dramatically in West Africa. Many areas of Liberia and Sierra Leone have passed tipping points in which ETUs can no longer accommodate the case load and measures are being implemented for community-based management. Bed capacity has reached a critical level in some areas. Novel but evidence-based strategies are desperately needed to streamline operations to meet this challenge. To reduce the community “viral load” and optimize use of limited bed and laboratory capacity, we recommend that convalescent patients should be discharged if asymptomatic for 48 hours and independently mobile, with appropriate counseling regarding possible infection risk and a basic discharge kit for infection control. This approach will have the additional benefit of enabling already constrained EVD testing laboratories to focus primarily on testing of new cases, thereby increasing the rate at which EVD PCR-negative suspect cases are identified and discharged, reducing the risk of ward-based EVD transmission to these individuals and freeing up additional beds. More conservative policies requiring blood or even urine to be reverse transcription-PCR (RT-PCR) negative before discharge may be maintained in centers and laboratories where capacity remains sufficient to perform such testing without unduly impacting the flow of patients. In the longer term, research is needed to establish evidence-based criteria and guidelines for safe discharge. This research must include collection of a wide range of bodily fluids from convalescent EVD patients and the testing of samples by both RT-PCR and, importantly, cell culture to detect infectious virus.
